# Depression and Reasoning Ability in Adolescents: Examining the Moderating Role of Growth Mindset

**DOI:** 10.3389/fpsyg.2022.636368

**Published:** 2022-03-14

**Authors:** Chen Hu, Cuicui Wang, Weiwei Liu, Daoyang Wang

**Affiliations:** ^1^College of Education, Hangzhou Normal University, Hangzhou, China; ^2^Department of Mental Health Education, School of Public Education, Zhejiang Institute of Economics and Trade, Hangzhou, China; ^3^Deqing Hospital of Hangzhou Normal University, Huzhou, China; ^4^Center for Cognition and Brain Disorders, The Affiliated Hospital of Hangzhou Normal University, Hangzhou, China; ^5^Institute of Psychological Sciences, Hangzhou Normal University, Hangzhou, China; ^6^Zhejiang Key Laboratory for Research in Assessment of Cognitive Impairments, Hangzhou, China; ^7^National Clinical Research Center for Mental Disorders, Department of Psychiatry, The Second Xiangya Hospital of Central South University, Changsha, China; ^8^Office of the Principal, Hangzhou Normal University, Hangzhou, China

**Keywords:** depression, reasoning ability, cognitive ability, growth mindset, adolescents

## Abstract

The present two-year longitudinal study aimed to examine the relationship between depression and reasoning ability in adolescents, and further investigated the modulation effect of growth mindset on this relationship. A total of 1,961 and 1,667 Chinese adolescents participated in the study for the first year (T1) and second year (T2), respectively. The results showed that T1 depression was negatively correlated with T1 growth mindset (*r* = −0.35, *p* < 0.001), T1 reasoning (*r* = −0.30, *p* < 0.001), and T2 reasoning (*r* = −0.23, *p* < 0.001). Regression analysis revealed that T1 depression and the interaction between T1 depression and T1 growth mindset significantly predicted T1 reasoning (β = −0.220/−0.044, all *p*s < 0.05). After controlling for gender, age, family socioeconomic status, and T1 reasoning ability, both T1 depression and the interaction between T1 depression and T1 growth mindset still significantly predicted T2 reasoning (β = −0.104/β = 0.054, all *p*s < 0.05). The simple slope analysis found that the negative correlation between depression and reasoning in the high growth mindset group was weaker than that of the low growth mindset group in both T1 and T2, suggesting that growth mindset plays a significant moderating role in the relationship between depression and reasoning. In conclusion, depression was negatively correlated with reasoning ability in adolescents, in addition, growth mindset moderated the relationship between depression and reasoning.

## Introduction

Depression refers to the mood disorder with a series of symptoms such as loss of interest, constant sadness, and impaired social function (Sadek and Bona, 2000). Studies revealed that depression could damage cognitive abilities such as attention, working memory, and inhibition. Besides, the depression could also increase the risk of behavioral problems, interpersonal conflict, and academic problems (Khesht-Masjedi et al., 2019). Adolescence is a critical stage with complex physical and mental transitions, and studies found that depressive symptoms among adolescents have gradually increased (Pimentel et al., 2020). A survey revealed that the incidence of depression in adolescents is 16–18% (Liu et al., 1999; Pimentel et al., 2020). Depression has become a common psychological problem among adolescents, however, compared with adults, depression among adolescents has not received enough attention from the public.

Reasoning ability refers to a form of thinking in which individual generalizes a general law in a specific situation or introduces a new conclusion based on existing judgments (Almomani et al., 2018). Reasoning ability is one of the most important cognitive skills (Tao et al., 2015). Studies revealed that depression leads to decreased cognitive ability (Baker and Channon, 1995; Liu and Wang, 2018). For example, individuals with depression are often accompanied by cognitive deficits, such as attention and decision-making difficulty (Liu and Wang, 2018). Depression has an adverse effect on reasoning ability in depressive patients (i.e., patients with clinical-level depression), for example, compared with healthy individuals, depressive patients have defects in abstract reasoning (Hecker et al., 2005). However, some studies revealed that the effect of depression on reasoning ability may be conditional. Parisi et al. (2014) found that depression did not significantly predicted reasoning ability for the elderly individuals, and the reason may be that there is a moderating variable between depression and reasoning ability (Parisi et al., 2014). Most previous studies focused on the depression patients or the elderly, and few studies investigated the effect of depression on reasoning ability for adolescents. Considering adolescents are at a critical stage for reasoning ability development (Demetriou et al., 2020), the present study aimed to evaluate the effect of depression on their reasoning ability and further explore possible moderating variables.

The growth mindset is the belief that attributes such as cognitive ability and personality are changeable, and the opposite belief is the fixed mindset which refers to the attributes are unchangeable (Dweck, 2006). Adolescents with growth mindset tend to regard setbacks and challenges as the opportunities to improve their abilities (Yeager and Dweck, 2012). Adolescents with growth mindset have more motivation to deal with challenging tasks such as reasoning. Researchers have found that adolescents with growth mindset have more positive motivation and better performance in solving reasoning problems (Sullivan and Davidson, 2014). Research has also shown growth mindset buffers the negative effects of stressful situations, depression, and anxiety (Molden et al., 2006; Job et al., 2010). Therefore, growth mindset may mediate the effect of depression on reasoning ability in adolescents. Till now, there are no research have been conducted to test whether and how growth mindset has a mediating effect on the adverse effects of depression on the reasoning ability for adolescents.

To further understand the effect of depression on reasoning ability for adolescents, the present study explored the relationship between depression and reasoning ability for two years. In addition, the present study examined whether growth mindset has a mediating effect on the effects of depression on adolescent’s reasoning ability. We hypothesized that depression in adolescents would be associated with reasoning ability, and that growth mindset moderates this relationship.

## Materials and Methods

### Participants

This longitudinal survey was carried out for two times: March 2016 (Time 1, T1) and March 2017 (Time 2, T2). The participants were selected from three secondary vocational schools in Guangzhou, China. Five classes were selected randomly at grade 1 and 2, respectively. Questionnaires in this research were collected online. Contemporary research showed that online questionnaires preformed good equivalence and similar psychometric properties to paper and pencil tests (Gosling et al., 2004; Aluja et al., 2007). The quality control questionnaire was used to provide a reliable quality control for online surveys. The questionnaire assessed the surrounding environment of the participants and their psychological feelings when answering the questions. It consists of four items as follows: “Are there urgency to handle with now? Such as doing homework or going out to play?” “How are you feeling now?” and “Please tell us the current noise situation in your surroundings?” (Wang and Liu, 2018). The participants who have the poor status that could not support them to give a reliable answer (such as having urgency to deal with) was excluded. Finally, there were 1,961 (Mean age = 16.97, SD = 1.10 years; male: n = 862, female: n = 1099) and 1,667 (Mean age = 16.84, SD = 0.94 years; male: n = 751, female: n = 916) adolescents participated in the study at T1 and T2, respectively. The differences for background variables between T1 and T2, as well as T2 and lost were shown in Table 1. Estimates of effect size were computed by using the Cohen”s d or Φ (small ≥ 0.20, medium ≥ 0.50, large ≥ 0.80) (Cohen, 1988). According to effect size, the difference in age between tracking and lost participants was significant (*t* = –6.72, *p* < 0.001, Cohen’s *d* = 0.43). The reason may be that the participants who in higher grade were lost in graduation.

**TABLE 1:** The differences for background variables between T1 and T2, as well as T2 and lost.

	Time 1 (T1)		Time 2 (T2)			Effect size	Time 2 (T2)		lost			Effect size
Gender	*N*	%	*N*	%	χ^2^	Φ	*N*	%	*N*	%	χ^2^	Φ
Male	862	43.96	751	45.05	0.44	–	751	45.05	111	37.76	5.11*	0.02
Female	1099	56.04	916	54.95			916	54.95	183	62.24		
	*M*	*SD*	*M*	*SD*	*t*	Cohen’s *d*	*M*	*SD*	*M*	*SD*	*t*	Cohen’s *d*
Age	16.97	1.10	16.84	0.94	3.79***	0.13	16.84	0.94	17.25	1.09	−6.72***	0.43
Family annual income	4.26	2.36	4.31	2.37	−0.73	−0.02	4.31	2.37	4.16	2.36	1.61	0.06
Father’s educational level	3.94	2.22	4.01	2.22	−1.05	−0.03	4.01	2.22	3.80	2.22	2.35*	0.10
Mother’s educational level	3.65	2.18	3.67	2.13	−0.34	−0.01	3.67	2.13	3.61	2.28	0.75	0.03

*Family annual income, 9 ≥ ¥200,000; 8 = ¥150,000–199,999; 7 = ¥100,000–149,999; 6 = ¥50,000–99,999; 5 = ¥30,000–49,999; 4 = ¥10,000–29,999; 3 = ¥6,000–9,999; 2 = ¥3,001–5,999; 1 ≤ ¥3,001; Father’s or mother’s educational level = the years of education for father or mother.*

*p < 0.05; **p < 0.01; ***p < 0.001.

All participants in this study were provided a complete explanation of the whole research. Parents/guardians of participants under 18 years old wrote informed content. The Ethics Board for Research Projects at Hangzhou Normal University reviewed and approved this research.

### Instruments

#### Background Questionnaire

The background questionnaire investigated three parts information includes gender, age, and family socioeconomic status through self-report form. The annual household income and the year of parents’ education as two indicators to represent the family socioeconomic status. The answer options of annual household income question as follows: 9 ≥ ¥200,000; 8 = ¥150,000–199,999; 7 = ¥100,000–149,999; 6 = ¥50,000–99,999; 5 = ¥30,000–49,999; 4 = ¥10,000–29,999; 3 = ¥6,000–9,999; 2 = ¥3,001–5,999; 1 = < ¥3,001.

#### The Center for Epidemiologic Studies-Depression Scale

The Center for Epidemiologic Studies Depression-Scale (CES-D) was developed by Radloff (1977) to assess depression. Four dimensions were included in the scale as follows: somatic and retarded activity, positive affect, depressed affect, and interpersonal. The scale contained 20 items, and each item was scored from 0 (rarely or none of the time) to 3 (most of the time). Four items in the dimension of positive affect were reverse coded to ensure the participants attending to each of the question and not answering carelessly. Aggregate scores for the CES-D range from 0 (never had depressive symptom) to 60 (most of the time had felt depressive symptom). The total scores greater or equal to 16 points was used to indicate depressive symptom (Radloff, 1991). Chen et al. (2009) had proved that the Cronbach’s α of CES-D is 0.88 for Chinese adults. The Cronbach’s α of the CES-D was 0.91 in the present study.

#### Growth Mindset Inventory

The Growth Mindset Inventory was developed by Dweck (2006). The Growth Mindset Inventory included two dimensions: fixed mindset (11 items) and growth mindset (9 items), totally 20 items. Answers were given based on 4-point Likert-type form (0 = totally disagree, 1 = disagree, 2 = agree, 3 = totally agree). The higher scores of the participants on either dimension of mindset, the more tendency with this mindset. The items of fixed mindset are reversely scored when calculated the total scores of this scale. Summed scores greater than 32 points indicates a higher tendency of growth mindset, in contrast, indicates fix mindset tendency (Mora, 2015). The Cronbach’s α of this inventory was 0.78 in the present study.

#### Reasoning Ability Test

**FIGURE 1:**
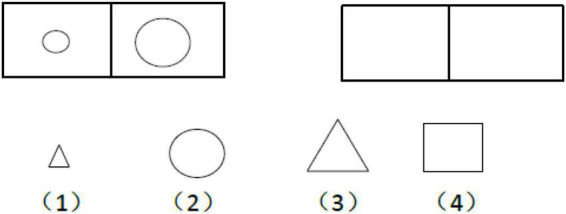
Graphic analogical reasoning test.

**FIGURE 2:**
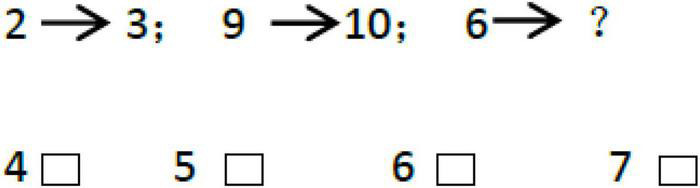
Digital analogical reasoning test.

**FIGURE 3:**
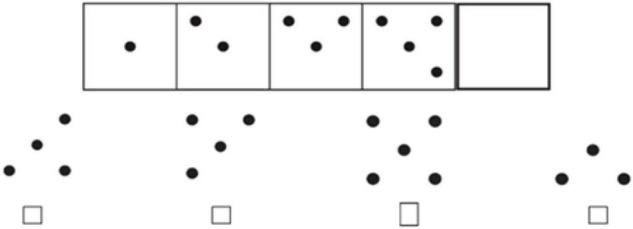
Graphic sequence inference task.

The Reasoning Ability Test was selected from the Investigation Report of Psychological Development Characteristics of Chinese Children and Adolescents by National Research Project Team (Dong and Lin, 2011). Reasoning Ability Test consisted of two subtests: inductive reasoning (18 items) and analogy reasoning (41 items), totally 59 items. Among them, the analogical reasoning ability included of both graphic analogical (e.g., Figure 1) and digital analogical reasoning (e.g., Figure 2) tasks, while the inductive reasoning ability only included graphic sequence inference task (e.g., Figure 3). The Cronbach’s α of each subtest and whole test are all beyond 0.70 in previous study (Dong and Lin, 2011). The internal consistency α coefficient of the scale was 0.89 in the present study.

### Statistical Analyses

SPSS 21.0 and PROCESS were used to analyze the data. All the variables were standardized before analysis. First, we conducted description and correlation analysis of all variables in the study. Second, regression analysis was used to examine the simultaneous moderation effect of T1 growth mindset on the relationship between T1 depression and T1 reasoning ability, and the moderation effect of T2 growth mindset on the relationship between T2 depression and T2 reasoning ability. In addition, we further examined the delayed moderation effect of the T1 growth mindset on the relationship between T1 depression and T2 reasoning ability. Third, a simple slope test was used to investigate the simultaneous and delayed moderation effect of growth mindset on the relationship between depression and reasoning ability.

The Harman’s single factor test was used to examine the common-method variance in the regression analysis. The report of the test showed that the number of factors that the eigenvalue greater than 1 were 14; the interpretation rate of the first factor was 35.06%, less than the critical criterion 40%. Therefore, the common-method bias did not exist in this study. It was acceptable for us to conduct regression analysis. Besides, Bootstrap was used to test whether the modulation effect was significant. The Bootstrap is a resampling method which was used to estimate statistics on participants by sampling a dataset with replacement, and this method has been widely used in previous studies (Tan and Holub, 2015). The number of resampling was 1,000 or 5,000. Bootstrap could alleviate the possibility of type I and type II errors effectively. This study used the Bootstrap method to construct 5,000 samples, each with a sample size of 1,667 participants. The 95% confidence interval (CI) was used in the present study. If the CI does not contain zero, it indicates the result was significant (Erceg-Hurn and Mirosevich, 2008). Estimates of effect size were computed by using the Cohen’s *d* or Φ (small ≥ 0.20, medium ≥ 0.50, large ≥ 0.80) (Cohen, 1988).

## Results

### Correlation Analysis

The descriptive statistics and correlations among gender, age, family’s socioeconomic status (annual family income and parents’ educational level), depression, reasoning ability, and growth mindset were shown in Table 2. The results showed that T1 depression was positively associated with mother’s educational level (*r* = 0.07, *p* = 0.001), and negatively associated with gender (*r* = −0.07, *p* < 0.001), T1 growth mindset (*r* = −0.35, *p* < 0.001), T1 reasoning ability (*r* = −0.30, *p* < 0.001), and T2 reasoning ability (*r* = −0.23, *p* < 0.001). T1 growth mindset was positively associated with gender (*r* = 0.07, *p* = 0.015), T1 reasoning ability (*r* = 0.29, *p* < 0.001), and T2 reasoning ability (*r* = 0.16, *p* < 0.001). In addition, T1 reasoning ability was positively associated with annual family income (*r* = 0.06, *p* = 0.001), and T2 reasoning ability (*r* = 0.41, *p* < 0.001).

**TABLE 2:** Demographic results and Pearson correlations between variables.

	M ± SD	1	2	3	4	5	6	7	8
1. Gender	–	–							
2. Age	16.97 ± 1.10	0.01	–						
3. Annual family income	4.26 ± 2.36	−0.07**	–0.01	–					
4. Father’s educational level	3.94 ± 2.22	–0.02	–0.01	0.17***	–				
5. Mother’s educational level	3.65 ± 2.18	−0.04*	−0.07***	0.17***	0.70***	–			
6. T1 Depression	22.57 ± 13.61	−0.07***	0.01	0.01	0.03	0.07**	–		
7. T1 Growth mindset	32.48 ± 4.33	0.07***	–0.02	0.02	0.01	–0.03	−0.35***	–	
8. T1 Reasoning ability	93.15 ± 13.96	0.04*	–0.02	0.06**	0.02	–0.03	−0.30***	0.29***	–
9. T2 Reasoning ability	90.00 ± 12.21	–0.01	0.01	0.01	–0.03	–0.02	−0.23***	0.16***	0.41***

*Gender: male = 0, female = 1; Family annual income, 9 ≥ 200,000; 8 = ¥150,000–199,999; 7 = ¥100,000–149,999; 6 = ¥50,000–99,999; 5 = ¥30,000–49,999; 4 = ¥10,000–29,999; 3 = ¥6,000–9,999; 2 = ¥3,001–5,999; 1 ≤ ¥3,001; Father’s or mother’s educational level = the years of education for father or mother.*

*p < 0.05; **p < 0.01; ***p < 0.001.

### Regression Analysis

Hierarchical regression analysis was used to test the predictive effect of depression on reasoning ability and the moderation effect of growth mindset. The depression was used as an independent variable, the growth mindset was used as the moderation variable, and reasoning ability were used as the dependent variable into the regression analysis (see Table 3).

**TABLE 3:** Regression analysis for the variables to predict Reasoning ability.

		T1 Reasoning ability			T2 Reasoning ability		
		*B*	*SE*	*t*	β	*SE*	*t*
Model 1	Gender	0.088	0.042	2.094*	−0.031	0.046	−0.677
	Age	−0.001	0.001	−1.339	0.001	0.001	0.759
	Annual family income	0.031	0.009	3.547***	−0.011	0.010	−1.095
	Father’s educational level	0.036	0.013	2.841**	−0.015	0.014	−1.116
	Mother’s educational level	−0.038	0.013	−2.881**	0.012	0.014	0.865
	T1 Reasoning ability	—	—	—	0.416	0.022	19.095***
		Δ*F* = 5.60***; Δ*R*^2^ = 0.01			Δ*F* = 60.87***; Δ*R*^2^ = 0.18		
Model 2	T1Depression	−0.220	0.020	-11.048***	−0.104	0.025	−4.211***
	T1Growth mindset	0.201	0.020	10.121***	0.011	0.023	0.461
		Δ*F* = 171.33***; Δ*R*^2^ = 0.12			Δ*F* = 10.44***; Δ*R*^2^ = 0.01		
Model 3	T1Depression × T1Growth mindset	−0.044	0.021	−2.083*	−0.054	0.025	−2.170*
		Δ*F* = 4.34*; Δ*R*^2^ = 0.01			Δ*F* = 4.71*; Δ*R*^2^ = 0.01		

*T1: N = 1961, T2: N = 1667.*

After controlling for gender, age, and family socioeconomic status, T1 depression has a significant negative predictive effect on T1 reasoning ability (β = −0.220, *p* < 0.001); T1 growth mindset has a significant positive predictive effect on T1 reasoning ability (β = 0.201, *p* < 0.001). The *R*^2^ change (Δ*R*^2^) between Model 2 and Model 3 was significant (Δ*R*^2^ = 0.01, *p* < 0.01). And the interaction between T1 depression and T1 growth mindset has a significant negative predictive effect on T1 reasoning ability (β = −0.044, *p* = 0.037), indicating that T1 growth mindset moderated the relationship between T1 depression and T1 reasoning ability. Therefore, growth mindset had the simultaneous moderation effect on the relationship between depression and reasoning ability in adolescents.

Further, after controlling for gender, age, family socioeconomic status, and T1 reasoning ability, T1 depression still negatively predicted T2 reasoning ability (β = −0.104, *p* < 0.001). The *R*^2^ change (Δ*R*^2^) between Model 2 and Model 3 was significant (Δ*R*^2^ = 0.01, *p* < 0.01). And the interaction between T1 depression and T1 growth mindset negatively predicted T2 reasoning ability (β = −0.054, *p* = 0.030), indicating that T1 growth mindset moderated the relationship between T1 depression and T2 reasoning ability. Therefore, growth mindset had a delayed moderation effect on the relationship between depression and reasoning ability in adolescents.

**FIGURE 4:**
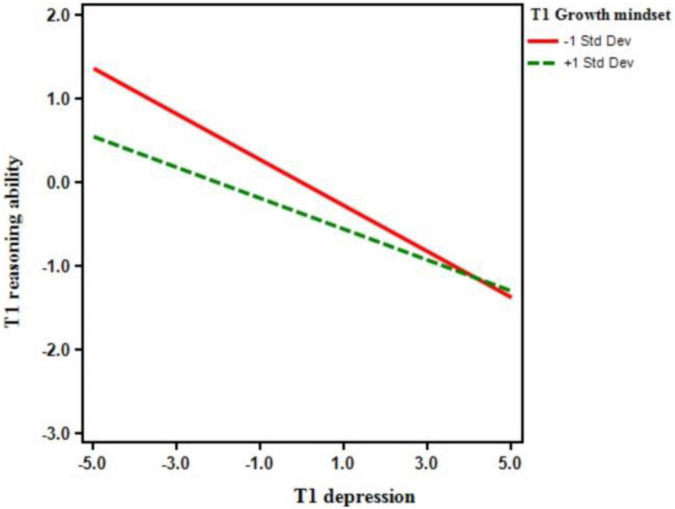
The moderation effect of T1 growth mindset on the relationship between T1 depression and T1 reasoning ability in adolescents.

To further investigate the moderation effect of growth mindset, a simple slope analysis was performed. The total points of Growth Mindset Inventory were divided into high and low level of groups based on the mean ± 1 standard deviation (Figure 4). We put reasoning ability as the dependent variable, depression as the independent variable. The results showed that T1 depression negatively predicted T1 reasoning ability in both T1 low level of growth mindset group (simple slope = −0.186, *t* = −7.138, *p* < 0.001) and T1 high level of growth mindset group (simple slope = −0.274, *t* = −8.413, *p* < 0.001). After further analysis, we found that the negative prediction effect between the depression and reasoning ability in the high level of growth mindset group was weaker than that of the low level of growth mindset group [*F*_(1_, _1929)_ = 12.01, *p* < 0.001, *f*^2^ = 0.15].

**FIGURE 5:**
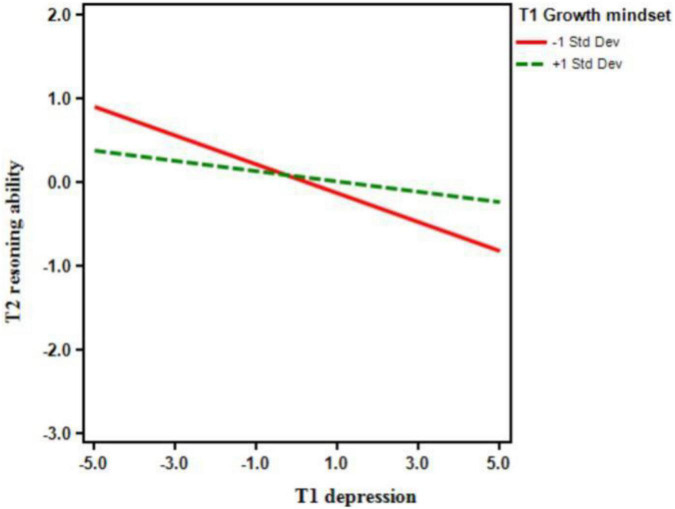
The moderation effect of T1 growth mindset on the relationship between T1 depression and T2 reasoning ability in adolescents.

Moreover, simple slope analysis was performed with T1 depression as an independent variable and T2 reasoning ability as the dependent variable. The results showed that T1 depression negatively predict T2 reasoning ability in both T1 low level of growth mindset group (simple slope = −0.061, *t* = −2.010, *p* = 0.031) and T1 high level of growth mindset group (simple slope = −0.173, *t* = −4.296, *p* < 0.001) (Figure 5). Therefore, the negative prediction effect of depression on reasoning ability in the high level of growth mindset group was weaker than that of the low level of growth mindset group [*F*_(1_, _1638)_ = 6.63, *p* < 0.001, *f*^2^ = 0.07].

## Discussion

The present two-year longitudinal study aimed to examine the relationship between depression and reasoning ability in adolescents, and further investigated the moderation effect of growth mindset on this relationship. Both the results of correlation and regression analysis showed that T1 depression not only negatively predicted T1 reasoning ability, but also negatively predicted T2 reasoning ability, indicating that the prediction effect of depression for reasoning ability was stable. Further, the simple slope analysis revealed that growth mindset could moderate the relationship between depression and reasoning ability at both simultaneous (T1) and delayed time (T2).

Depression has an adverse effect on reasoning ability in adolescents, which was consistent with previous studies (Radenhausen and Anker, 1988; Hecker et al., 2005). Radenhausen and Anker (1988) found that the performance of reasoning was worse for individuals with depressive emotion than that of positive and neutral emotions, indicating that depression may have an adverse effect on reasoning. Hecker et al. (2005) also revealed that compared with healthy individuals, depressive patients have defects in abstract reasoning. Reasoning requires the flexible allocation of attentional resources to synthesize the content in working memory into new conclusions. However, depression could take up the limited resources of working memory to process negative information, which hinders the reasoning (Ellis and Ashbrook, 1987). Studies revealed that depressive patients cannot effectively coordinate the cognitive functions of attention and working memory in complex reasoning tasks (Silberman et al., 1983; von Hecker et al., 2005).

Most previous studies have focused on depressive patients (Hecker et al., 2005) or the elderly (Parisi et al., 2014), and the present study extended the previous studies. Adolescents face academic challenges and changes in both physical and mental, and therefore, the depressive mood has become a common mental health problem in adolescents. The results of the present study help us to understand that depression has a negative effect on reasoning in adolescents, which indicates that educators and parents should pay more attention to the mental health of adolescents.

This two-year longitudinal study found that growth mindset moderated the relationship between depression and reasoning ability at both simultaneous and delayed time. Therefore, growth mindset buffered the relationship between depression and reasoning. Growth mindset allows individuals to focus on how to improve their abilities through effort in challenging tasks and stressful situations (Molden et al., 2006). Job et al. (2010) found that under the condition of self-exhaustion, individuals with growth mindset have better self-control performance. The cognitive ability, especially reasoning ability, is critical for the academic performance in adolescents. Consequently, growth mindset moderated the relationship between depression and reasoning for adolescents. Educators could consider growth mindset intervention to reduce the negative impact of depression on cognitive function for adolescents.

There are some limitations in this research. First, adolescents’ participants are all from secondary vocational schools. Future research could enrich age groups and school types of samples and further investigate whether the growth mindset still moderates the relationship between depression and reasoning in academic high school. Second, the present study did not investigate the effect of growth mindset intervention, and future studies could further examine this question. Such research could further investigate the specific mechanism by which growth mindset can weaken the adverse effects of depression on reasoning. Third, the present study did not investigate the influence of reasoning to depression. Our future studies would further explore the effect of reasoning ability on depression.

## Data Availability Statement

The datasets presented in this article are not readily available because the present study was funded by the agencies that did not authorize the disclosure of normative data. Requests to access the datasets should be directed to the National Social Science Fund of China and DW, daoyang@ahnu.edu.cn.

## Ethics Statement

The studies involving human participants were reviewed and approved by Institutional Review Board of Human Research Ethics Committee at Hangzhou Normal University. Written informed consent to participate in this study was provided by the participants’ legal guardian/next of kin.

## Author Contributions

CH designed the research programme, conducted data analysis, wrote the original manuscript, and revised the manuscript. CW contributed to the data collection, data interpretation, and manuscript writing and amendment. WL contributed to the manuscript writing, amendment, and publication funds. DW supervised the entire study, ensure the accuracy and integrity definition of intellectual content in the manuscript, and revised the manuscript. All authors contributed to the article and approved the submitted version.

## Conflict of Interest

The authors declare that the research was conducted in the absence of any commercial or financial relationships that could be construed as a potential conflict of interest.

## Publisher’s Note

All claims expressed in this article are solely those of the authors and do not necessarily represent those of their affiliated organizations, or those of the publisher, the editors and the reviewers. Any product that may be evaluated in this article, or claim that may be made by its manufacturer, is not guaranteed or endorsed by the publisher.

## References

[B1] Almomani F. Al-momani M. Alsheyab N. Mhdawi K. A. (2018). Reasoning abilities and potential correlates among Jordanian school children. *Matern. Child Health J.* 22 501–511. 10.1007/s10995-017-2416-7 29282593

[B2] Aluja A. Rossier J. Zuckerman M. (2007). Equivalence of paper and pencil vs internet forms of the ZKPQ-50-CC in Spanish and French samples. *Pers. Individ. Differ.* 43 2022–2032. 10.1016/j.paid.2007.06.007

[B3] Baker J. E. Channon S. (1995). Reasoning in depression: impairment on a concept discrimination learning task. *Cogn. Emot.* 9 579–597. 10.1080/02699939508408984

[B4] Chen Z. Y. Yang X. D. Li X. Y. (2009). Psychometric features of CES-D in Chinese adolescents. *Chin. J. Clin. Psychol.* 17 443–448.

[B5] Cohen J. (1988). *Statistical Power Analysis for the Behavioral Sciences*, 2nd Edn. Hillsdale, NJ: Erlbaum.

[B6] Demetriou A. Kazi S. Makris N. Spanoudis G. (2020). Cognitive ability, cognitive self-awareness, and school performance: from childhood to adolescence. *Intelligence* 79:101432. 10.1016/j.intell.2020.101432

[B7] Dong Q. Lin C. D. (2011). *Standardized Tests in Children and Adolescent Mental Development in China.* Beijing: Science Press.

[B8] Dweck C. S. (2006). *Mindset: The New Psychology of Success.* New York, NY: Ramdom House.

[B9] Ellis H. C. Ashbrook P. W. (1987). “Resource allocation model of the effects of depressed mood states on memory,” in *Affect, Cognition and Social Behavior*, eds Fiedler K. Forgas J. (Gottingen: Hogrefe), 25–43. 10.1037//0096-3445.126.2.131

[B10] Erceg-Hurn D. M. Mirosevich V. M. (2008). Modern robust statistical methods: an easy way to maximize the accuracy and power of your research. *Am. Psychol.* 63 591–601. 10.1037/0003-066X.63.7.591 18855490

[B11] Gosling S. D. Vazire S. Srivastava S. John O. P. (2004). Should we trust web-based studies? A comparative analysis of six preconceptions about internet questionnaires. *Am. Psychol.* 59:93104. 10.1037/0003-066X.59.2.93 14992636

[B12] Hecker U. V. Sedek G. Engle R. W. Mclntosh D. N. (2005). *Cognitive Limitations in Aging and Psychopathology.* Cambrige: Cambridge University Press.

[B13] Job V. Dweck C. S. Walton G. M. (2010). Ego depletion–is it all in your head? Implicit theories about willpower affect self-regulation. *Psychol. Sci.* 21 1686–1693. 10.1177/0956797610384745 20876879

[B14] Khesht-Masjedi M. F. Shokrgozar S. Abdollahi E. Habibi B. Asghari T. Ofoghi R. S. (2019). The relationship between gender, age, anxiety, depression, and academic achievement among teenagers. *J. Fam. Med. Prim. Care* 8 799–804. 10.4103/jfmpc.jfmpc_103_18 31041204 PMC6482750

[B15] Liu J. L. Wang L. (2018). Cognitive dysfunction and brain mechanisms in major depression disorder. *Chin. Sci. Bull.* 63 1973–1983. 10.1360/N972018-00129

[B16] Liu X. C. Ma D. D. Kurita H. Tang M. Q. (1999). Self-reported depressive symptoms among Chinese adolescents. *Soc. Psychiatry Psychiatr. Epidemiol.* 34 44–47. 10.1007/s001270050110 10073120

[B17] Molden D. C. Plaks J. E. Dweck C. S. (2006). “Meaningful” social inferences: effects of implicit theories on inferential processes. *J. Exp. Soc. Psychol.* 42 738–752. 10.1016/j.jesp.2005.11.005

[B18] Mora I. A. (2015). Capturing the best skills to generate and inspire the multigenerational workforce. *J. Bus. Manage. Stud.* 1 1–9.

[B19] Parisi J. M. Franchetti M. K. Rebok G. W. Spira A. P. Carlson M. C. Willis S. L. (2014). Depressive symptoms and inductive reasoning performance: findings from the ACTIVE reasoning training intervention. *Psychol. Aging* 29 843–851. 10.1037/a0037670 25244465 PMC4316212

[B20] Pimentel F. D. Della C. P. Patias N. D. (2020). Victims of bullying, symptoms of depression, anxiety and stress, and suicidal ideation in teenagers. *Acta Colomb. Psicol.* 23 230–240. 10.14718/acp.2020.23.2.9

[B21] Radenhausen R. A. Anker J. M. (1988). Effects of depressed mood induction on reasoning performance. *Percept. Mot. Skills* 66 855–860. 10.2466/pms.1988.66.3.855 3405709

[B22] Radloff L. S. (1977). The CES-D scale: a self-report depression scale for research in the general population. *Appl. Psychol. Meas.* 1 385–401. 10.1177/014662167700100306 26918431

[B23] Radloff L. S. (1991). The use of the center for epidemiologic studies depression scale in adolescents and young adults. *J. Youth Adolesc.* 20 149–166. 10.1007/BF01537606 24265004

[B24] Sadek N. Bona J. (2000). Subsyndromal symptomatic depression: a new concept. *Depress. Anxiety* 12 30–39. 10.1002/1520-6394200012:13.0.CO;2-P 10999243

[B25] Silberman E. K. Weingartner H. Post R. M. (1983). Thinking disorder in depression: logic and strategy in an abstract reasoning task. *Arch. Gen. Psychiatry* 40 775–780. 10.1001/archpsyc.1983.01790060073009 6860078

[B26] Sullivan P. Davidson A. (2014). “The role of challenging mathematical tasks in creating opportunities for adolescent reasoning,” in *Proceedings of the 37th Annual Meeting of the Mathematics Education Research Group of Australasia*, Sydney, NSW.

[B27] Tan C. C. Holub S. C. (2015). Emotion regulation feeding practices link parents’ emotional eating to children’s emotional eating: a moderated mediation study. *J. Pediatr. Psychol.* 40 657–663. 10.1093/jpepsy/jsv015 25770312

[B28] Tao S. Liu H. Y. Zhou C. M. Wang C. C. Sun C. Y. Xu F. (2015). The roles of school psychological environment in grades 4-6 students’ cognitive development: a multilevel analysis of the national representative data. *J. Psychol. Sci.* 142 267–286.

[B29] von Hecker U. Sedek G. Piber-Dabrowska K. Bedynska S. (2005). “Generative reasoning as influenced by depression, aging, stereotype threat, and prejudice,” in *Cognitive Limitations in Aging and Psychopathology*, eds Engle R. W. Sedek G. von Hecker U. McIntosh D. N. (New York, NY: Cambridge University Press).

[B30] Wang D. Y. Liu Z. G. (2018). Testing measurement invariance of satisfaction with life scale (SWLS) based on the multi – group confirmatory factor analysis. *Psychol. Explor.* 80–85. 10.1007/s11136-018-1922-4 29926346

[B31] Yeager D. S. Dweck C. S. (2012). Mindsets that promote resilience: when adolescents believe that personal characteristics can be developed. *Educ. Psychol.* 47 302–314. 10.1080/00461520.2012.722805

